# Breaking bad news: what parents would like you to know

**DOI:** 10.1136/archdischild-2019-318398

**Published:** 2020-10-30

**Authors:** Marije A Brouwer, Els L M Maeckelberghe, Agnes van der Heide, Irma M Hein, Eduard A A E Verhagen

**Affiliations:** 1 Department of Pediatrics, University Medical Center Groningen, Groningen, Netherlands; 2 Institute for Medical Education, University Medical Center Groningen, Groningen, Netherlands; 3 Department of Public Health, Erasmus Medical Center, Rotterdam, Zuid-Holland, Netherlands; 4 Department of Psychiatry, Academic Medical Center, Amsterdam, North Holland, Netherlands

**Keywords:** comm child health, palliative care, patient perspective, qualitative research

## Abstract

**Objective:**

Breaking bad news about life-threatening and possibly terminal conditions is a crucial part of paediatric care for children in this situation. Little is known about how the parents of children with life-threatening conditions experience communication of bad news. The objective of this study is to analyse parents’ experiences (barriers and facilitators) of communication of bad news.

**Design:**

A qualitative study consisting of a constant comparative analysis of in-depth interviews conducted with parents.

**Setting:**

The Netherlands.

**Participants:**

Sixty-four parents—bereaved and non-bereaved—of 44 children (aged 1–12 years, 61% deceased) with a life-threatening condition.

**Interventions:**

None.

**Results:**

Based on parents’ experiences, the following 10 barriers to the communication of bad news were identified: (1) a lack of (timely) communication, (2) physicians’ failure to ask parents for input, (3) parents feel unprepared during and after the conversation, (4) a lack of clarity about future treatment, (5) physicians’ failure to voice uncertainties, (6) physicians’ failure to schedule follow-up conversations, (7) presence of too many or unknown healthcare professionals, (8) parental concerns in breaking bad news to children, (9) managing indications of bad news in non-conversational contexts, and (10) parents’ misunderstanding of medical terminology.

**Conclusions:**

This study shows healthcare professionals how parents experience barriers in bad news conversations. This mainly concerns practical aspects of communication. The results provide practical pointers on how the communication of bad news can be improved to better suit the needs of parents. From the parents’ perspective, the timing of conversations in which they were informed that their child might not survive was far too late. Sometimes, no such conversations ever took place.

**What is already known on this topic?**

- Communication of bad news receives insufficient attention in medical training.
- Physicians often feel uncomfortable in delivering bad news.
- Current studies—mostly limited to palliative care—show that parents are often not satisfied with the communication about their child’s life-threatening prognosis.

**What this study adds?**

- This study shows 10 themes (barriers and facilitators) perceived by parents with regard to bad news conversations.
- From the experiences of parents we deduced practical advice on how to improve bad news conversations.
- The most important message: talk with parents, even when prognosis is uncertain.

## Introduction

During their years of medical training, physicians are instructed about how to deliver bad news to patients and their families.^1 2^ Communicating bad news—in this article defined as conversations between physicians and parents concerning their child’s severe diagnosis, limited treatment options or poor prognosis—is especially difficult in paediatrics. Here, it must navigate the triangular relationship between the healthcare professionals, the parents and the child.^3^ Communication can have a positive or negative effect on their parents’ perceptions of the decision-making process.^4 5^ However, many healthcare professionals feel uncomfortable when delivering bad news.^6 7^

The few studies that have focused on parents’ experiences found a general lack of satisfaction with the way in which bad news is communicated.^4 8–14^ However, current knowledge suffers from two limitations. First, many studies focus on oncology,^4 10–15^ and second, they mainly focus on children who already receive palliative care, which in practice means that they mainly include children with a terminal diagnosis.^8–10 13–15^ Yet not all children with life-threatening conditions receive palliative care, or have been diagnosed with a terminal prognosis.^16–18^ It is important to understand how communication of bad news concerning children with life-threatening conditions might be improved to better suit the needs of parents and children. This article provides a focused qualitative analysis of parental experiences of communication of bad news, and is part of a larger qualitative interview study into care and decision-making for children (aged 1–12 years) with life-threatening conditions.^19^

## Methods

In a large-scale, nationwide qualitative study, we interviewed parents on care and decision-making for young children (aged 1–12 years). The themes on communication of bad news presented in this paper are the results of a focused analysis of parents’ experiences.

### Sample

We recruited bereaved and non-bereaved parents of children (aged 1–12 years) with a life-threatening condition. Life-threatening conditions are here defined as all medical conditions that are potentially lethal and/or life limiting. Parents were excluded when their child had died more than 5 years prior to the interview. Recruitment was tailored to yield maximum variety in terms of condition, age, cultural background, level of education and place of residence. Recruitment continued until data saturation was achieved. First indications of thematic saturation were observed after 30 interviews,^20^ and extra interviews (12) were conducted to ascertain maximum variety.

### Recruitment

Study participants were recruited in the period from November 2016 to October 2018. Parent support groups used their online platforms to reach potential participants, and paediatricians and paediatric palliative care teams were contacted to invite potential participants. All potential participants received full information about the study, were given an opportunity to ask questions before participation and gave their written consent.

### Interviews

A single face-to-face, in-depth interview was held with parents, usually at their place of residence (average duration: 2 hours). The topic guide is added as an online supplemental file 1 to this article. The interviews were held in Dutch, recorded on audio media and subsequently transcribed verbatim. Interviews were conducted by the first author, MAB (female, MA, PhD student), who had undergone formal training for this purpose. The participants involved had no prior relation with the interviewer, nor were they offered any form of remuneration. Parents were free to choose to be interviewed alone or together. Emotional support from a social worker was offered after the interview, but none of the participants used this option.

10.1136/archdischild-2019-318398.supp1 Supplementary data

### Analysis

The aim of the analysis was to provide a qualitative description of barriers in communication of bad news as perceived by parents.^21^ A constant comparative analysis was used.^20^ For the purposes of this article, we analysed those sections of the interviews that concerned the communication of bad news.

The first author coded the transcripts in terms of communication-related content. All of the authors read the selected material to familiarise themselves with the content. Themes were identified by a reiterative process of comparing and contrasting interview sections, which were further specified using *Atlas.ti*, a software program for coding qualitative texts.^22^ Coding was performed by the first two authors, and reviewed by all authors. Any discrepancies were discussed until consensus was achieved. The coding scheme is added as an online supplemental file 2.

10.1136/archdischild-2019-318398.supp2 Supplementary data

Regular meetings with an advisory group of parents, researchers and paediatricians were held to discuss the results and translate them into recommendations of care.

## Results

We held interviews (n=42) with 64 parents of 44 children with a life-threatening condition, 24 of them bereaved. All of the children involved suffered from a variety of life-threatening conditions. Parents were recruited from all parts of the Netherlands. Every Dutch academic medical centre, as well as over 20 local hospitals, was represented. Details of participants’ characteristics are shown in tables 1 and 2.

**Table 1:** Children’s characteristics (n=44)

Gender	
Male	20 (45.5%)
Status	
Living	17 (38.6%)
Deceased	27 (61.4%)
Age*	
1–3	15 (34.0%)
4–6	8 (18.2%)
7–9	9 (20.5%)
10–12	12 (27.3%)
Diagnosis	
Malignancies	18 (40.9%)
Congenital malformations	17 (38.6%)
Cardiovascular	4 (9.1%)
CNS	3 (6.8%)
Other	1 (2.2%)
Undiagnosed	1 (2.2%)
Physical abilities†	
Unimpaired or mildly impaired	9
Moderately impaired	15
Severely impaired	20
Cognitive abilities†	
Unimpaired or mildly impaired	18
Moderately impaired	7
Severely impaired	19
Chance of dying‡	
Low chance of dying	10
Significant chance of dying	23
Terminal prognosis	11

*For deceased children, the age at death.

†Based on the interviews, we estimated average abilities from the moment of the diagnosis until the moment of the interview, or until the start of the terminal phase.

‡Based on the interviews, we estimated the chance of dying at the moment the bad news was delivered. Where several conversations were involved, we averaged the known prognosis.

CNS, central nervous system.

**Table 2:** Parents’ characteristics (n=42 parent couples, a total of 64 parents were interviewed)

Interviewed parent (n=42)	
Both parents (interviewed together)	22 (52.4%)
Mother alone	20 (47.6%)
Father alone	0 (0.0%)
Relationship status (n=42)	
Married/together	34 (81.0%)
Level of education, mothers (n=42)	
Low educational level	1 (2.4%)
Middle educational level	15 (35.7%)
Higher educational level	14 (33.3%)
University education	12 (28.6%)
Level of education, fathers (n=42)	
Lower educational level	4 (9.5%)
Middle educational level	15 (35.7%)
Higher educational level	14 (33.3%)
University education	9 (21.4%)
Ethnicity of parents, according to participant (n=42)	
Dutch	36 (85.7%)
Mixed	6 (14.3%)
Religious/spiritual beliefs (n=64)	
None	39 (60.9%)
Christian	19 (29.7%)
Other	6 (9.4%)
Family composition (n=42)	
1 child	8 (19.0%)
2 children	22 (52.4%)
3 children	10 (23.8%)
4 or more children	2 (4.8%)

The experiences of parents included both facilitators and barriers, but parents were most explicit about the barriers to good communication of bad news. Conversations about the (possible) death of their child were most prominent in their narratives, but their experiences on bad news conversations included other information (such as the severity or treatability of the condition) as well. We identified 10 themes: (1) a lack of (timely) communication, (2) physicians’ failure to ask parents for input, (3) parents feel unprepared during and after the conversation, (4) a lack of clarity about future treatment, (5) physicians’ failure to voice uncertainties, (6) physicians’ failure to schedule follow-up conversations, (7) presence of too many or unknown healthcare professionals, (8) parental concerns in breaking bad news to children, (9) managing indications of bad news in non-conversational contexts, and (10) parents’ misunderstanding of medical terminology.

### Theme 1: a lack of (timely) communication

During the illness of their child, some parents seemed to be unaware that their child might not survive during treatment. We identified four types of prognosis: (1) conditions with a terminal prognosis, (2) conditions with a gradually changing prognosis (such as certain oncological conditions), (3) conditions with an all-or-nothing prognosis (such as cardiac surgeries) and, finally, (4) conditions where no precise diagnosis could be made (this was the case with several children with unknown metabolic conditions). Especially in the last two categories, there was little or no discussion of the possibility of death.

You just don’t know what you are getting into. And looking back, I do think physicians knew, I think they realized quite quickly what was happening. But we did not. Because they never told us. Maybe they didn’t withhold the information on purpose, I don’t know, but… (M37)

Interviewer: Looking back, do you think you would have wanted to know?

Mother: Yes, when I look at that entire period, I would have wanted to know it. Maybe not on day 1, but at least a lot sooner. Then we might have done things differently that final year.

Several parents recalled retrospectively that they had never been explicitly told that their child’s future was uncertain or that their child might die, until shortly before the moment of death.

I went to the doctor, and asked, ‘Is he going to be okay?’ He replied, ‘well, with the right…’ And then I said, ‘No, I really want to know. I’m asking you.’ And it turned out that there was no chance at all. That’s when I said, ‘Then I don’t want all this treatment for him any more. We should take him home.’ After that it went quickly: he died the following day. (M20)

In some cases, a conversation never took place because parents were referred to another hospital and physicians assumed that the conversation had taken place. Parents felt that it was important to have open conversations about the child’s uncertain prognosis. They said that parents would be thinking about this anyway, and not mentioning it created a taboo. They felt that it should not be up to parents to take the initiative in such matters, as that would make them feel that they were giving up on their child.

You have to have the courage to [talk about the possibility of death]. And we were lucky to have one doctor who had that courage. I think a lot of doctors find that really difficult, because of who they are, and because of their training. (…) But it helps to talk, or philosophize together about death. And of course there are limits to what is possible, but there are possibilities as well, and it helps to be open about how you feel towards those. (F09)

The failure to hold such conversations leaves parents unprepared when the message is finally delivered, and deprives them of any opportunity to make decisions about their future.

If they had told me, I would have taken him home instead, to give him a dignified end of life. (M20)

### Theme 2: failure to ask parents for input

Parents appreciated conversations in which they were treated as equals. They emphasised that, in conversations of this kind, they wanted physicians to take them seriously when they signalled symptoms or evaluated the child’s quality of life. Physicians only see small snippets of a child’s life. This makes their evaluation incomplete and serves to underline the importance of a parental perspective.

They only see [our daughter] during check-ups in the hospital. But she behaves completely differently there. And then they draw all kinds of conclusions about how she is doing, and I always feel, yes, but when we get home, everything will be different again. (M38)

Recognising that some people might want more information than others, some parents advised physicians to ‘ask parents how they wish to be informed—Whether they want all medical information or not’ (M04).

### Theme 3: parents feel unprepared during and after the conversation

Parents stated that they often felt overwhelmed by the conversation, because they were not sufficiently prepared for the conversation. One mother explained that she was given the news that her daughter’s tumour was terminal while she was lying in a hospital bed, recovering from a caesarean. She had been given no prior notification of this conversation, and felt overwhelmed and bereft of autonomy.

The feeling of lying there, in your pyjamas, looking up to all these doctors, has left such a bad taste in my mouth. And I remember wondering, does it really need to be done like this? (M01)

The importance of facilities to support parents following conversations was also emphasised. Several participants remember walking out of the bad news conversation and being unable to find a quiet space where they could calm down and call their family, and with no idea of how to get home safely in that distressed state of mind.

Suddenly we were in the main hall again, and we said to each other, ‘What are we supposed to do now? I think we should call some people?’ We had come to the hospital by car, so I said, ‘I don’t think I can drive home right now.’ My husband said, ‘I think I can drive…’ But having to do that, that’s just irresponsible! (M23)

### Theme 4: a lack of clarity about future treatment

The parents emphasised that, when people are informed that their child’s illness is incurable, they should be told what to expect in terms of care and support.

[After the bad news conversation] we went home, [with the message] ‘go and make good memories with her.’ But how? I don’t think we had another appointment, or anything. For two weeks after that conversation, we had no idea what to do: Where should we go now? Who should we call? What should we do? We had to find out all those things ourselves. (M21)

Other parents had more positive experiences, appreciating that physicians promised to be there for them.

What I really appreciated was that besides the information about prognosis, they also said, this is what we can do for you. (…) We are going to support you through what is to come. (M15)

### Theme 5: physicians’ failure to voice uncertainties

Parents felt that physicians often found it difficult to talk freely in situations involving uncertainty. Conversations were postponed until the details had been confirmed; in others, information that was presented as factual later turned out to be incorrect.

Just tell us that you don’t know. (F01)

Honesty about physicians’ lack of certainty was appreciated.

[The doctor] said: ‘Are you okay with a second opinion? Because I really don’t know how to proceed at the moment.’ And I said, ‘I’m just happy that you honestly admit not knowing it, even as an expert on this.’ (M03)

### Theme 6: failure to schedule follow-up conversations

Several parents said that, after receiving bad news, they were immediately expected to ask questions and make decisions. This gave them no time to process the news.

We were sitting there, in front of 14 white coats, and they said, ‘We have seen a cerebrovascular accident, what do you want?’ That was the first thing they asked. Without any context. (M07)

Several parents recommended that bad news conversations be carried out in two stages. The first stage would involve delivering the bad news, while the second stage would give parents an opportunity to ask questions, or to discuss decisions. A few parents had experienced such two-stage bad news conversations and appreciated this approach.

[A physician can] check during the second meeting, ‘did they hear everything I told them?’ Because maybe they only absorbed part of the message. (F31)

### Theme 7: presence of too many or unknown healthcare professionals

In several cases, bad news conversations included a group of physicians, many of whom were complete strangers to the parents. Parents would have preferred a more intimate setting.

Six or seven people came in (…) and then they told us the news. And I just thought, ‘Why are all these people here? What is the value of that? How am I supposed to react?’ Everyone is looking at you, and of them, we only knew the neurologist. It was really uncomfortable and overwhelming. (M01)

In cases where presence of several healthcare professionals was required, parents advised to introduce them and explain their presence at the conversation.

### Theme 8: parental concerns about how bad news should be broken to their child

Most of the parents in the study were positive about the way in which bad news had been broken to their child. However, some parents disagreed with physicians about how much children should be told about their illness. Others felt abandoned when it came to discussing illness and death with a young child. Several participants remarked that much of the information provided was targeted at children with terminal oncological conditions. Children themselves also had an impact on communication: some flatly refused to talk about their illness, while others were actively involved.

She would always know when the results of the MRI would come back, and would pick up the phone when the doctor called. She would say, ‘Oh, you can tell me!’ And the doctor would have a conversation with her about it. (F25)

### Theme 9: managing indications of bad news in non-conversational contexts

During the treatment, important messages were sometimes inadvertently conveyed by other means: notes on a hospital bed, the waiting time before the results arrived or overheard conversations. Parents stressed that healthcare professionals need to be aware of the impact of such messages.

We were notified that the MRI had been rescheduled because the neurologist was hesitant to wait so long. The planners just make a schedule and notify you. But for us it was an all-important message. So it would have been nice to have a little more… compassion there. (M15)

### Theme 10: parents’ misunderstanding of medical terminology

Parents often felt that they understood what had been explained to them by healthcare professionals, but not always. An example of the latter is provided by a couple explaining that they had never realised that the brain tumour of their daughter was in fact, a cancer… Medical terminology may not carry the same meaning for healthcare professionals as for parents, creating misunderstandings.

## Discussion

The aim of this study was to investigate parents’ experiences (barriers and facilitators) in communication of bad news. We studied this on a uniquely broad scale, with a large number of participants, nationwide, and a representation of various life-threatening conditions.^23^

The narratives of parents gave insight in the various experiences of parents during communication of bad news. Some of the experiences of this group corroborate findings from earlier studies, such as the need for empathic communication,^8–10 24 25^ and the importance of timely conversations about prognosis.^6 18 26 27^ This study shows that especially for children with uncertain prognosis (which is often true with neurological and metabolic conditions) bad news conversations were often absent.

The study adds insight in how lack of conversation impedes decision-making. Decisions that influence life expectancy occur long before the illness is terminal. Parents expressed that they would have made different decisions if more information about the child’s prognosis had been provided. This finding underlines the urgency to hold timely conversations about decision-making in line with parental needs for individualised care planning.^28^

Parents specifically mentioned the need for honesty regarding their child’s prognosis, as an opportunity to discuss the proportionality of treatment and possible end-of-life decisions. A lack of information could limit parents’ ability to make well-informed decisions about their child’s quality of life (including end-of-life decisions). In some instances, the healthcare professionals involved may feel that the parents are not ready to hear the prognosis.^15 28^ However, they may be more prepared for the bad news than the medical staff suspect. More research is needed but, for the time being, the most pragmatic approach may be the one put forward by the parents in the study—‘Ask parents how they wish to be informed’.

Parents’ wish to be seen as an equal partner in communication about bad news ties in with ongoing changes in physician–patient relationships, in which the classic paternalistic model is giving way to models of shared decision-making.^29 30^ Implementation in paediatrics remains limited.^31^ Lack of information is a frequent barrier in paediatric shared decision-making,^31^ but in palliative decision-making, crucial information originates from parents. The parents’ intimate knowledge of their child can—and should—complement the healthcare professional’s technical/medical expertise, necessitating communication. This is particularly true of conversations about quality of life and suffering. Although, in the experiences of parents in our study, this equal partnership is not always achieved, it is comforting to see that initiatives to enhance shared decision-making in paediatric palliative care are being developed.^32^

Our study does have some limitations. First, it focused on young children (aged 1–12), so the results may not be generalisable to adolescents or neonates. Second, cultural differences in decision-making may mean that the experiences of Dutch parents differ from parents in other countries. Third, we focused on the issues involved from the parents’ perspective, which means that we cannot be certain how the bad news was delivered. However, people’s experiences are central to their lives, and improving communication is, in the end, about how communication is understood.

## Conclusion

This study shows how parents perceive the communication of bad news. Their experiences highlight two main points. First, the experiences of parents mainly concern very ‘practical’ aspects of communication: where conversations happen, who is present, how they are scheduled. Second, from the parents’ perspective, communication of bad news often took place far too late. Indeed, in some cases, no such conversations ever took place.

**Figure 1:**
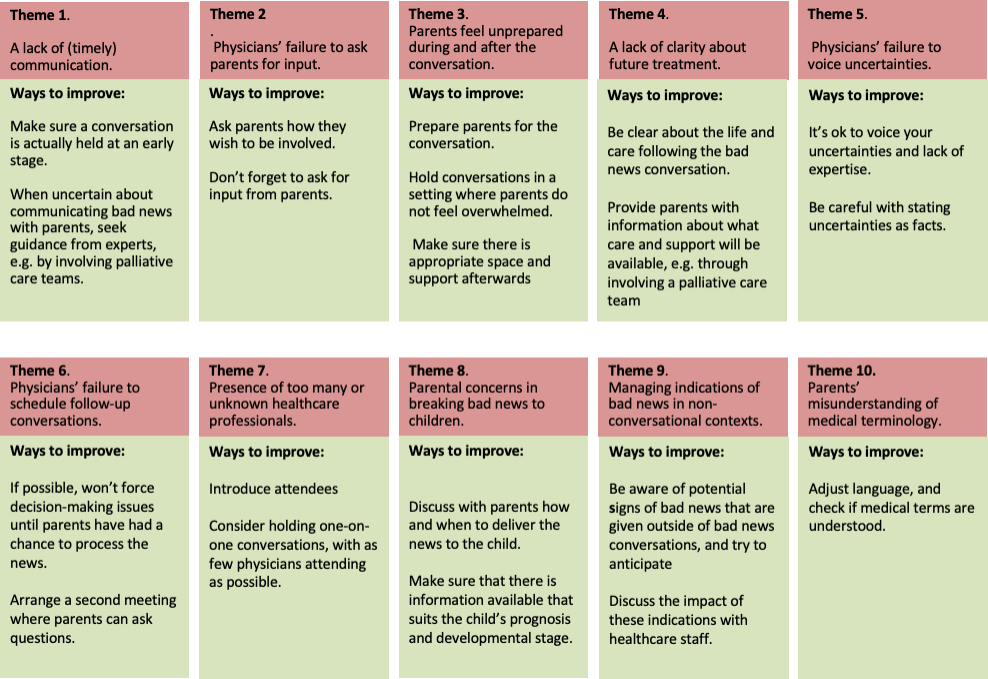
Ten practical ways to improve communication of bad news, based on parents' experiences.

This study shows that parents experience a significant number of barriers in the communication of bad news. Their experiences, however, provide an opportunity to improve communication about life-threatening conditions, for example, by being aware that adequate information is disclosed to parents, especially in circumstances of uncertain knowledge. Parents express a need to be informed, even if their child’s situation is uncertain. This may be anticipated by having conversations shortly after a condition is classified as life threatening or by asking parents how much they want to know. Second, parents may be better prepared for the conversation by creating circumstances that empower, rather than overwhelm them—for example, by having regular one-to-one conversations. Together with our advisory group we translated the themes into a list of advises to improve communication of bad news. This list is presented in figure 1.

Good communication matters. It influences good care,^14–16^ and when parents voice dissatisfaction about their children’s care, this tends to be about communication, rather than the purely medical aspects of care.^33 34^ By studying the ways in which parents perceive communication of bad news, we can learn how to improve the way in which we communicate when caring for children with life-threatening conditions. Above all, we need to remind ourselves to talk to parents about the future of their child, especially when the prognosis is uncertain.
