# Supplementary figures and images for: Breaking bad news: what parents would like you to know
Source: Arch Dis Child. 2020 Oct 30;106(3):276–81. doi: 10.1136/archdischild-2019-318398 (PMC7907584; doi:10.1136/archdischild-2019-318398)

Appendix: Topic guide

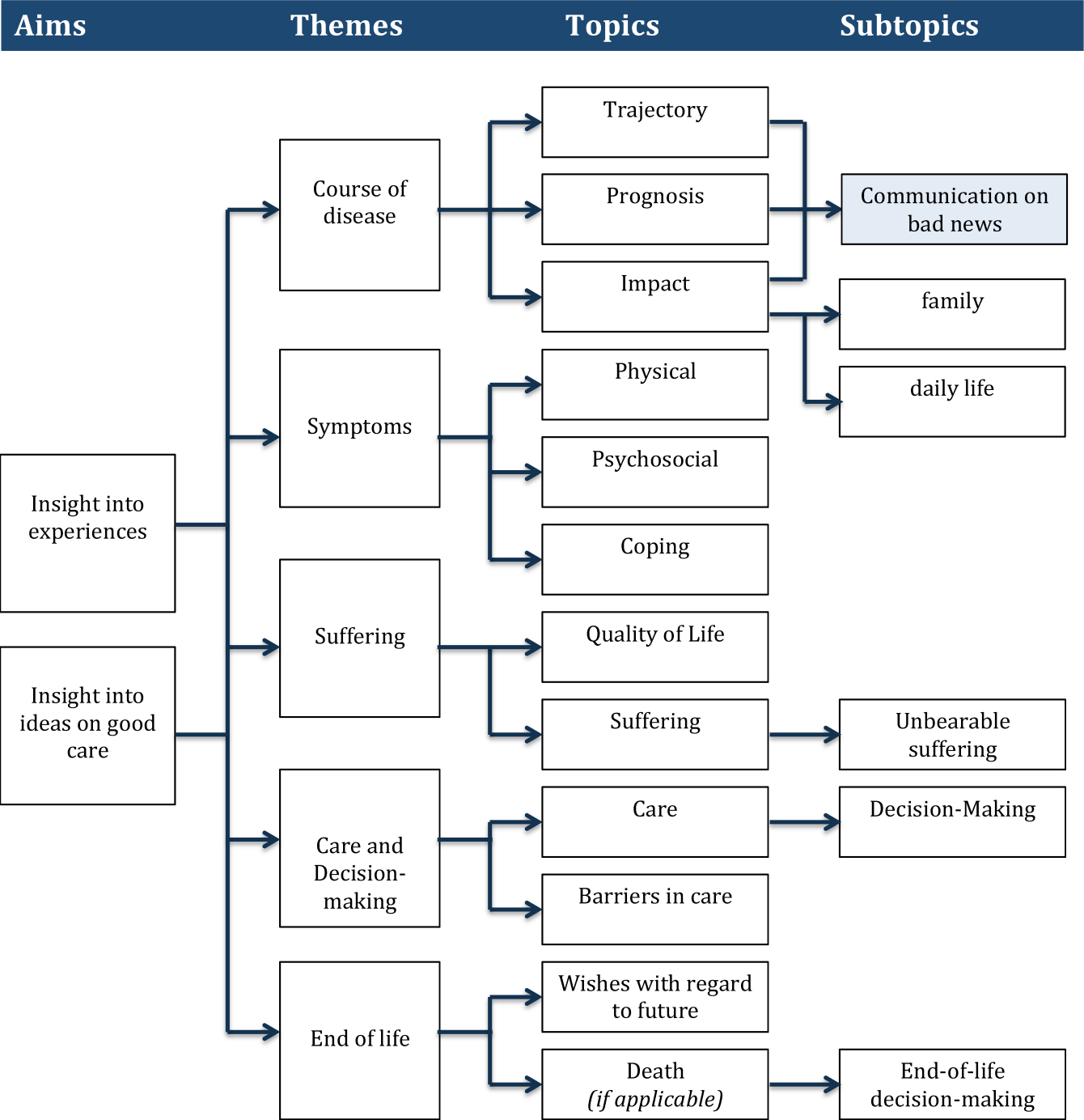

Supplement: Supplementary data [file archdischild-2019-318398supp001.pdf]

Coding Scheme Bad News Communication

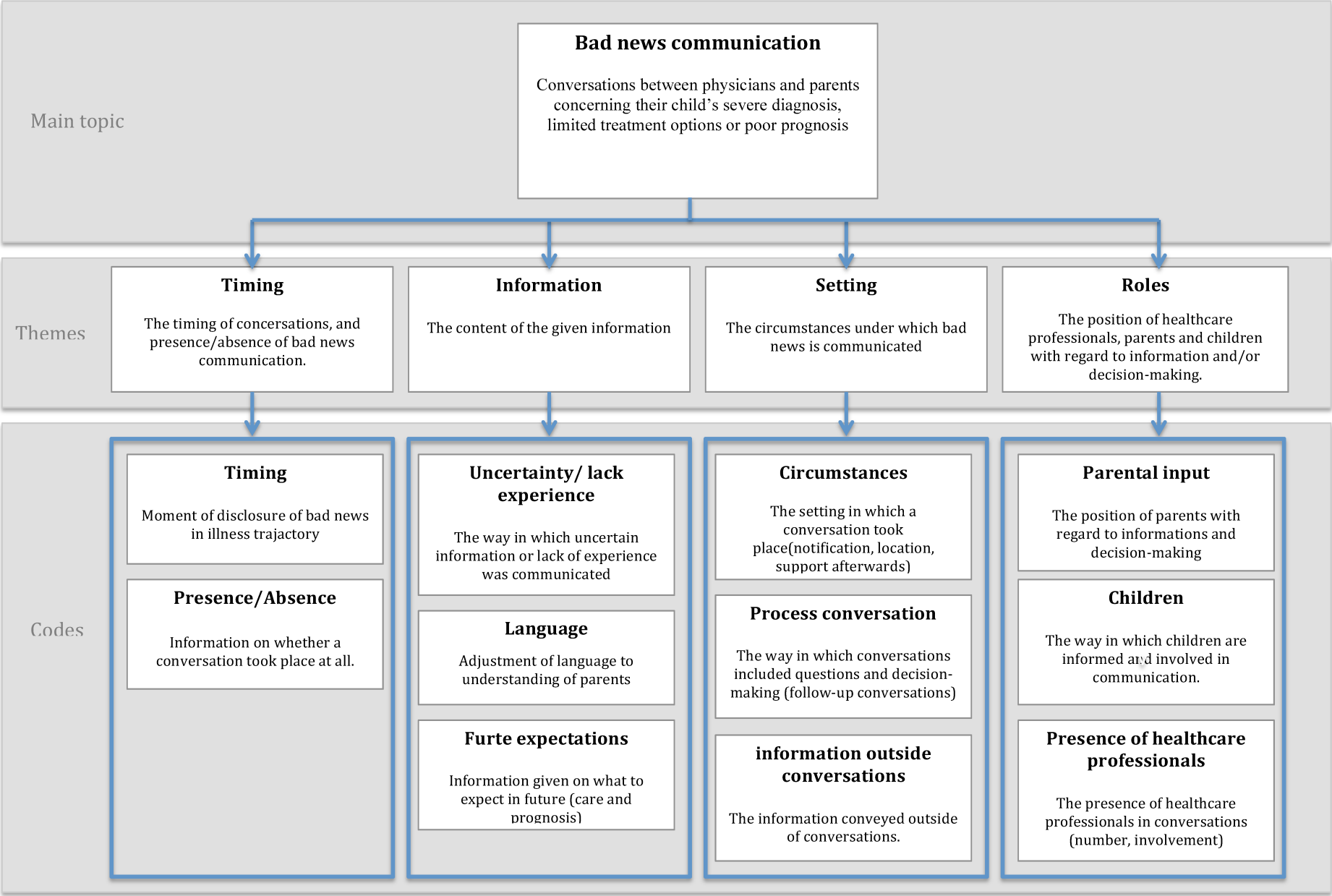

Supplement: Supplementary data [file archdischild-2019-318398supp002.pdf]
